# Surgical Approaches and Oncological Outcomes in the Management of Duodenal Gastrointestinal Stromal Tumors (GIST)

**DOI:** 10.3390/jcm10194459

**Published:** 2021-09-28

**Authors:** Nikolaos Vassos, Aristotelis Perrakis, Werner Hohenberger, Roland S. Croner

**Affiliations:** 1 Division of Surgical Oncology, Department of Surgery, Mannheim University Medical Center, University of Heidelberg, 68167 Mannheim, Germany; 2 Department of Surgery, University Hospital Erlangen, University of Erlangen-Nuremberg, 91054 Erlangen, Germany; werner.hohenberger@uk-erlangen.de; 3 Department of Surgery, University Hospital Magdeburg, 39106 Magdeburg, Germany; aristotelis.perrakis@med.ovgu.de (A.P.); roland.croner@med.ovgu.de (R.S.C.)

**Keywords:** gastrointestinal stromal tumors, GIST, duodenum, surgery, imatinib

## Abstract

Background: Duodenal gastrointestinal stromal tumors (GIST) are a rare subset of GIST. Their surgical management in this anatomically complex region consists of varied approaches, and the administration of imatinib mesylate (IM) has not been clarified. Methods: We retrospectively reviewed patients with duodenal GIST treated during a 10-year-period. We analysed the clinicopathological characteristics and survival factors and evaluated the perioperative and long-term outcomes based on the extent of resection ((ocal-resection (LR) versus pancreaticoduodenectomy (PD)) and the IM-administration. The median follow-up period was 60 months (range, 12–140). Results: A total of thirteen patients (M:F = 7:6) with median age of 64 years (range, 42–77) underwent resection of duodenal GIST. Median tumor size was 5.2 cm (range, 1.5–13.3). Eight patients (61.5%) underwent LR and five patients (38.5%) PD. R0-resection was achieved in 92.5%. Neoadjuvant IM-therapy was administered in five patients leading to tumor downsizing and in 40% to less-extended resection. The PD group consisted of larger tumors with higher mitotic count, mostly located in D2 (*p* = 0.031). The PD group had longer operative time (*p* = 0.026), longer hospital stay (*p* = 0.016), and higher rate of postoperative complications (*p* = 0.128). The actuarial 1-, 3-, and 5-year overall survival were 92.5%, 84%, and 73.5%, respectively, whereas the disease-free survival rates at 1, 3, and 5 years were 91.5%, 83%, and 72%, respectively. A tendency towards increased risk of disease recurrence was demonstrated for patients with tumor >5 cm and high-risk potential. There was not statistic survival benefit for one or the other surgical approach. Conclusion: The type of resection depends on duodenal site of origin and tumor size. LR can be the treatment of choice for duodenal GIST whenever technically feasible. Recurrence of duodenal GIST is dependent on tumor biology rather than surgical approach. Administration of IM in neaodjuvant setting should be considered in cases with high-risk GIST scheduled for PD since it might facilitate less-extended resection.

## 1. Introduction

Duodenal gastrointestinal stromal tumors (GIST) compromise a rare subset of tumors with an overall frequency of 3–5% of GIST but make up 30% of primary duodenal tumors of each entity [1,2,3,4]. Most cases are sporadic, but 5% occur in the context of a familial syndrome (i.e., neurofibromatosis type 1, Carney triad) [5,6,7]. Patients with duodenal GIST usually present with abdominal pain due to obstruction, anemia, or gastrointestinal bleeding, but small duodenal GISTs may be incidental findings [7].

During the past years, a huge amount of knowledge has been gained regarding the biology and clinical behavior of GIST, and the management of GIST has dramatically altered as a consequence of these achievements [5,8,9]. However, given that duodenal GIST are rare, the characteristics, prognosis, and optimal management have not been well clarified. More studies in the literature addressing the topic of duodenal GIST are limited by case reports [1,10,11,12,13,14,15] and small study populations [7,16,17,18,19,20,21,22,23,24,25,26]. More rarely, there are larger single-/multi-institutional experiences [27,28,29,30,31,32,33,34,35].

Surgery remains the gold standard treatment for patients with primary non-metastatic duodenal GIST [16,36,37]. However, the surgical treatment of duodenal GIST is challenging and remains to be established due to peculiar anatomic location and direct proximity to important neighboring anatomic structures (pancreatic head, ampulla of Vater, common bile duct, mesenteric root). This has resulted in choice of different surgical procedures according to the tumor presentation without standardized rules. A number of authors have reported various procedures ranging from limited resection (LR), such as wedge local resection, segmental duodenectomy, and pancreas-sparing duodenectomy to pancreaticoduodenectomy (PD).

Nowadays, the treatment of both primary and metastatic GIST has been rapidly evolved with the development of specific molecular targeted [38,39,40,41,42]. The potential of tyrosine-kinase-inhibitor (TKI) therapy in neoadjuvant or adjuvant setting for duodenal GIST has not widely been explored. Therefore, a comprehensive risk assessment with regard of patient outcome is necessary to compare the beneficial effects of limited or major surgery and the impact of neoadjuvant or adjuvant TKI therapy for duodenal GIST.

The purpose of this study was to evaluate the role of surgery in the management of duodenal GIST comparing the perioperative and long-term oncological outcome of each surgical procedure (LR vs. PD) and the effect of ΤΚΙ therapy determining factors associated with prognosis.

## 2. Materials and Methods

### 2.1. Patient Selection and Data

Patients who underwent a surgical resection of a GIST at our institution during a ten-year period were retrospectively reviewed. Among a total of 120 GIST patients, fifteen patients with duodenal GIST were enrolled in this study. The clinicopathological data of these patients were collected from our sarcoma database of the Department of Surgery, University Hospital Erlangen, and were summarized in a retrospective analysis. Following data were retrieved: sex, age, clinical presentation, tumor characteristics, anatomical location of the tumor (first (D1), second (D2), third (D3), or fourth (D4) portion of the duodenum), tumor size, mitotic count, risk classification, surgical procedure, resection status, postoperative morbidity and mortality, (neo)adjuvant TKI therapy, patterns of recurrence, and follow-up details.

### 2.2. Tumor Characteristics

Tumor size was defined as the greatest dimension of the tumor in the surgical specimen or the dimension at the radiological imaging in case of preoperative ΤΚΙ therapy. Tumors were detected via endoscopy and/or endoscopic ultrasound by performing biopsies. When a biopsy via endoscopy was not feasible, a computed tomography (CT)-guided biopsy was performed. Pathological diagnosis of GIST was confirmed in all cases according to histological and immunohistochemical work-up. Immunohistochemical staining with CD117, DOG-1, CD34, smooth-muscle actin, desmin, S-100, and Ki67 was performed. Mitoses were counted in 50 high-power fields (HPF). The risk category was defined based on tumor size, mitotic count, and tumor location according the consensus guidelines of the National Institutes of Health (NIH-NCI) workshop [8] and the updated Armed Forces Institute of Pathology (AFIP) criteria published by Miettinen and Lasota [9]. Mutation analysis of KIT exons 9, 11, 13, and 17 as well as Platelet Derived Growth Factor Receptor Alpha (PDGFRA) exons 12, 14, and 18 was performed using direct sequencing of PCR products and recorded when available.

### 2.3. Operative and Therapeutic Characteristics

According to the preoperative imaging and depending on the size and the location of the tumor (i.e., distance from the ampulla of Vater, involvement of the head of pancreas), a limited or extended resection was performed. Limited resection (LR) included either wedge local resection with primary closure or segmental duodenectomy with end-to-end anastomosis. Extended resection included a pylorus-preserving partial pancreaticoduodenectomy (PD) according to Traverso-Longmire [43]. Resection margin status was defined as R0 (macroscopically complete resection with surgical margins free of microscopic disease), R1 (macroscopically complete resection with positive microscopic surgical margins), and R2 (macroscopically incomplete resection) [44]. Postoperative complications were classified using the Clavien-Dindo classification [45] and the post-operative pancreatic fistula (POPF) according to the International Study Group on Pancreatic Fistula classification [46]. Postoperative mortality was defined as death occurring during the hospital stay or as a consequence of a postoperative complication.

The indication for pre- and postoperative TKI therapy was given at the discretion of the multidisciplinary sarcoma board. Tumor response was assessed every 3 months according to Response Evaluation Criteria in Solid Tumors (RECIST) as a complete response (CR), partial response (PR), stable disease (SD), or progressive disease (PD) [47].

### 2.4. Follow Up

Follow-up parameters were measured from the date of surgery. Assessment for follow-up was made using clinical examination, thoraco-abdominal CT, endo-ultrasonography, and, eventually, pancreatic magnetic resonance imaging (MRI). Disease relapse was defined as local recurrence or distant metastases. Particularly, local recurrence was defined as recurrent disease in the region of the previously located tumor and metastasis as disease in distant sites predominantly liver and peritoneum. The median follow-up of patients was 60 months (range, 12–140 months).

### 2.5. Statistical Analysis

Statistical analysis was performed by using the SPSS version 21.0 (SPSS Inc., Woking, UK). Comparisons of clinicopathologic characteristics between surgical groups were assessed using the chi-square test for dichotomous and categorical variables. The study endpoints were disease-free survival (DFS) and overall survival (OS). DFS and OS were calculated on the basis of the interval from the date of surgical resection to the date of clinical or radiological evidence of disease relapse, last follow-up, or death, whichever occurred first. Survival curves were plotted using the Kaplan–Meier method [48] and differences between groups were compared by log-rank tests [49]. Cox proportional hazard models were used to estimate hazard ratios for DFS and to determine independent risk factors [50]. All statistical tests were two-sided, and *p* < 0.05 was considered statistically significant.

## 3. Results

### 3.1. Clinicopathological Data

Fifteen patients with duodenal GIST were reviewed. All but two patients underwent surgical treatment, and a total of thirteen patients were included in our study. There were seven men and six women, and the median age at presentation was 64 (range, 42–77) years. There were twelve symptomatic patients; in one patient, the duodenal GIST was an incidental finding. The most common presentation of symptomatic duodenal GIST was abdominal pain followed by gastrointestinal bleeding. Two patients had a history of neurofibromatosis. The duodenal GIST were located in the second (*n* = 5; 38.5%), third (*n* = 5; 38.5%), and fourth portion of duodenum (*n* = 3; 23%). The majority of patients (*n* = 14, 94%) presented with solitary disease, and in one patient, the disease was multifocal. The tumor size on cross-sectional imaging ranged from 1.5 to 13.3 cm (median, 5.2), and the tumors were described as well circumscribed or encapsulated, sharply demarcated without infiltrative growth. Using the Fletcher classification scheme, three tumors had very low, three had low, three had intermediate, and four high risk for aggressive behavior. The risk of recurrence according to Miettinen resulted in three cases with no risk, three cases of low risk, and the remaining cases (*n* = 7) with high-risk potential. The patient and tumor characteristics are listed in Table 1.

### 3.2. Surgical Details and Postoperative Course

**Figure 1:**
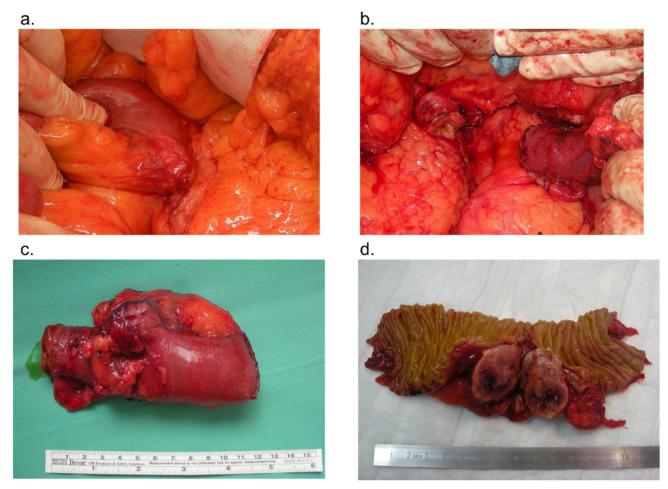
Intraoperative finding of duodenal GIST originating from the third portion of duodenum (**a**). Limited resection of duodenal GIST in terms of segmental duodenectomy (**b**). Specimen of duodenal GIST (**c**). Macroscopic appearance of duodenal GIST with outward growth and a centrally ulcerated umbilication (**d**).

Extensively, of the 13 patients with duodenal GISTs, 8 (61.5%) underwent LR, while 5 (38.5%) underwent PD. Among the patients who underwent LR, a wedge resection was performed in four patients and a segmental duodenectomy with primarily duodenojejunostomy reconstruction in four patients (Figure 1). Lymphadenectomy was performed in patients who underwent a PD, but no positive lymph nodes were detected. Postoperative course after LR in all patients was uneventful. Postoperative morbidity was only recorded in two patients of the PD group. One of them suffered from passage disturbance, which was treated conservatively, and the other patient experienced a grade IIIa complication in terms of pancreatic fistula, which was treated with interventional radiological techniques. Postoperative mortality was not recorded.

Resection with R0 status was achieved in the vast majority of patients (*n* = 12, 92.5%). One patient of the PD group was noted to have microscopic disease at the margin. Histologically, eleven tumors were of spindle cell differentiation, and two tumors were of mixed-type differentiation combining spindle cell and epithelioid areas. Immunohistochemically, all duodenal GIST (100%) were positive for KIT (CD117), and ten tumors (77%) expressed CD34, too. The mitotic rate per 50 HPFs ranged from 0 to 150 /50HPFs (median; 15). The mitotic count was less than five mitoses/50 high-power fields (HPF) in ten cases and more than five in three cases. Results of mutational analysis were available for 12 patients. Mutations in exon 11 were most frequently found (*n* = 7), whereas mutations in exon 9 and wild-type tumors were found in four and one patients, respectively.

### 3.3. Comparison between Patients Treated by LR and PD

Comparison of clinical data between patients of LR and PD group showed no significant differences in gender, age, or symptoms. Interestingly, bleeding was only present among patients of LR group. Patients with tumors located in the second part of duodenum were mostly treated by PD (PD: 80% vs. LR: 12.5%, *p* = 0.031) because of the close distance to ampulla of Vater or even involvement of pancreatic head. Patients who ultimately underwent PD were also more likely to present with a larger tumor (PD: 6.1 cm vs. LR: 4.7 cm; *p* = 0.489) of a higher mitotic count (*p* = 0.314). As expected, PD was significantly associated with a longer operative time (*p* = 0.026) and a longer duration of hospital stay (*p* = 0.016), whereas patients who received PD had a higher rate of post-operative complications than LR (*p* = 0.128). More details are demonstrated in Table 1.

### 3.4. Imatinib Mesylate

In our cohort, five patients were treated with neoadjuvant TKI therapy (400 mg/d) for a median length of 6.5 months (range, 5–9) before surgery. Interestingly, neoadjuvant TKI therapy was more frequently administered to patients affected by tumors arising in the second portion. Stable disease was observed in three patients and partial response according to RECIST criteria in two patients resulting in decreasing the extent of resection and performing LR instead of the scheduled PD. Adjuvant imatinib was administered to five patients (PD, *n* = 2/5 vs. LS, *n* = 3/8) who had high-risk GIST. Four patients received both neo- and adjuvant imatinib.

### 3.5. Follow-Up and Prognostic Analysis

Survival data could be obtained for all patients, and no patient was lost to follow-up. The median follow-up period of the patients was 60 months (range, 12–140). During follow-up, no patient developed local recurrence. Four patients (two from LR group and two from PD group) developed distant metastasis after a median disease-free interval of 28.5 months (range 0–70). Site of distant metastases was liver in all four cases, combined in two cases with osseous or peritoneal metastasis. Specifically, of the two patients of LR group, one patient developed synchronous hepatic and osseous metastases, and one developed metachronous hepatic and peritoneal metastases 20 months after operation. The two patients of PD group developed metachronous hepatic metastases 24 and 70 after the operation, respectively. As expected, none of the GIST with low or no risk for malignancy according to Miettinen’s criteria developed progression. In contrary, four of the cases (57%) with high risk of malignancy revealed disease progression in terms of metastatic disease. Similarly, none of the GIST patients with a tumor size <5 cm developed metastases; all patients with metastatic disease had an initial tumor size of >5 cm.

**Figure 2:**
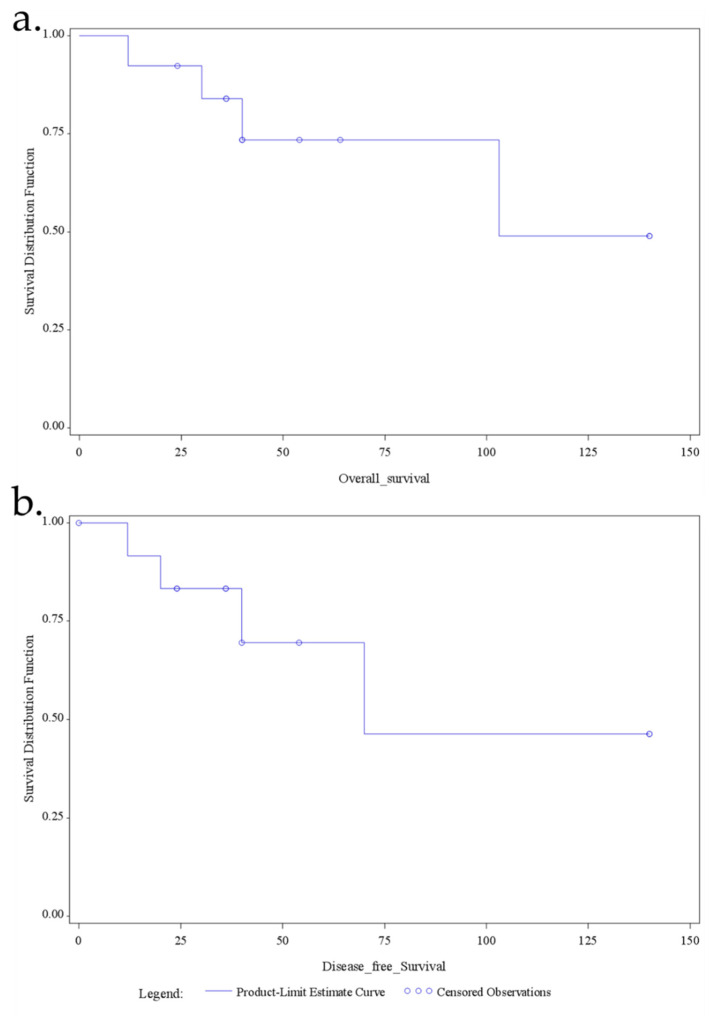
Kaplan–Meier estimator shows the overall survival (**a**) and the disease-free survival (**b**) for the patients with duodenal GIST.

**Figure 3:**
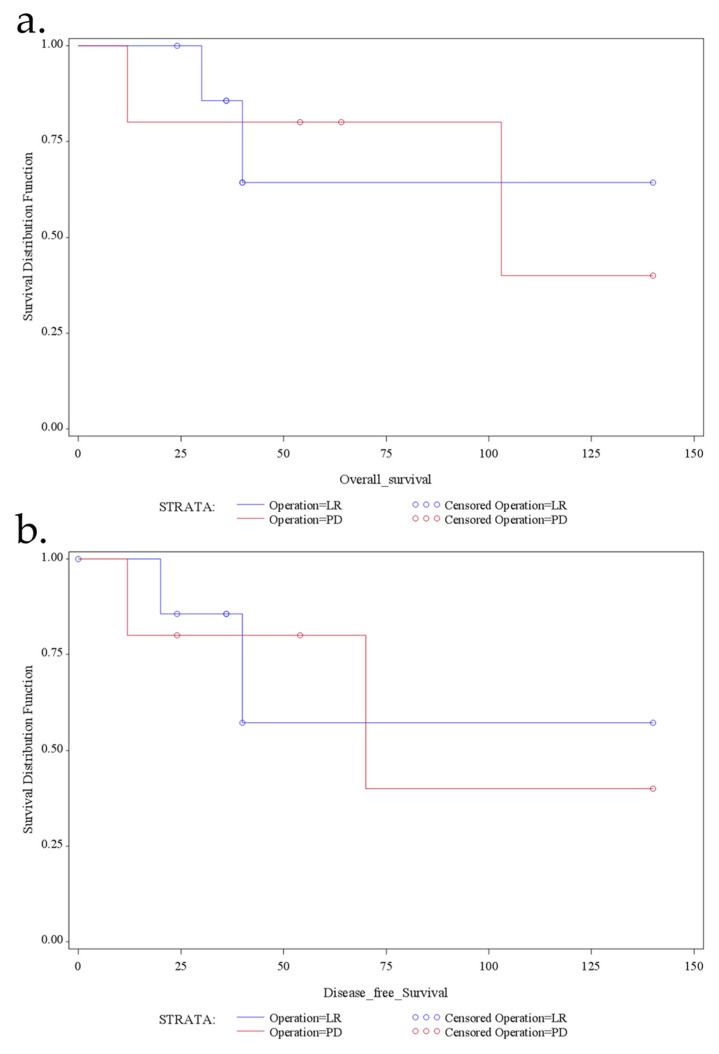
Kaplan–Meier estimator shows the overall survival (**a**) and the disease-free survival (**b**) for the patients with duodenal GIST and comparing LR and PD.

Overall survival and disease-free survival are demonstrated in Figure 2, with a mean overall survival of 60 months (median, 40; range, 12–140) and a mean disease-free survival of 49 (range, 12–140) months. Specifically, the overall 1-, 3-, and 5-year actuarial survival was 92.5%, 84%, and 73.5%, respectively, and the DFS 1-, 3-, and 5-year actuarial survival was 91.5%, 83%, and 72%, respectively. Besides, there was no significant survival benefit for one or the other surgical approach regarding overall survival (*p* = 0.209) or disease-free survival (*p* = 0.461) (Figure 3).

Univariable and multivariable analysis showed that no clinical or histopathologi-cal parameter was statistically associated with the overall survival. Regarding DFS, the only significant predictor for disease recurrence was the age >50 years, whereas there was an obvious tendency towards increased risk of disease recurrence for patients with high-risk potential tumors and tumors >5 cm with high mitotic count compared to patients with low-risk potential, smaller tumors with low mitotic count; however, due to the small patient number, the findings were not statistically significant.

## 4. Discussion

GIST of different anatomical sites not only vary in morphology and gene expression but also in clinical presentation and clinical outcome [8,9]. The characterization of the different subsets of GIST defining tailored management strategies is of great importance. In this analysis, we focused on a cohort of duodenal GIST. We identified thirteen patients who represent 10% of all GIST patients treated at our institution, which is above the previous studies, where GIST of duodenum mostly represent only 3–5% of all GIST [1,4,5]. Duodenal GIST can be located in all four parts of the duodenum but mostly involve the second one [1,4,27,28]. Our study showed a distribution in almost all parts of duodenum, and we did find a higher incidence of lesions in both the second and third portion.

The clinical presentation of duodenal GIST is highly variable depending on the size, location, and the existence of mucosal ulceration [1,4,7]. Most tumors present with abdominal pain and/or gastrointestinal bleeding and more rarely with intestinal or biliary obstruction [1,4,7,17,28]. Small tumors without mucosal ulceration are usually asymptomatic representing incidental findings. Only one small tumor of 18 mm was asymptomatic in our series and was incidentally found in endoscopic retrograde cholangiopancreatography for cholecystolithiasis. We noted that patients who ultimately required a PD were more likely to present with larger tumors that caused pain. In contrast, patients who ultimately required LR more often presented with bleeding.

The pathological and immunohistochemical features of duodenal GIST are different compared with gastric and small intestinal cases [1]. Duodenal GIST are relatively smaller in size in contrast to a median size of gastric and small intestinal GISTs of 6 to 7 cm, respectively [1]. The mean tumor size of 5.2 cm in the present study is in accordance with previous findings in other studies [4]. Furthermore, they usually have a low mitotic count (<5/50 HPF) in comparison to GIST of other localizations [1]. In our series, 77% of our patients suffered from GIST with low mitotic count, which was also reported by Miettenen et al. and Winfield et al., (72% and 75%, respectively) [4,7]. The biological appearance of GISTs shows a great variance. Comparing size and mitotic index in various locations in the gastrointestinal tract, duodenal GIST appear to have a relatively high risk of recurrence [1,4]. Interestingly, no patient of our cohort developed local recurrence but distant metastases (metastatic rate: 31%); specifically, only the high-risk cases developed disease progression (4/7 cases) as compared to none of the patients with low-risk GIST (0/6).

Surgical resection with the achievement of microscopically negative margins and the avoidance of tumor rupture is the therapy of choice for primary localized and non-metastatic duodenal GIST [8]. Unlike other gastrointestinal carcinomas, wide margins and regional lymphadenectomy are not required because GIST are mostly well encapsulated tumors without tendency for local invasion and hematogenous spread occurring only rarely if ever and associated with lymphatic infiltration [51,52,53]. We did not perform a lymphadenectomy in our cases of LR group, and in the cases of PD group, no lymph node metastases were detected. Even the largest clinicopathologic series of duodenal GIST did not demonstrate lymphatic spread in 167 patients [4]. Furthermore, in the present study, no lymph node recurrence has been detected on follow-up.

Various techniques of resection for duodenal GIST have been advocated. In addition to extended resection, surgical options, which entail a more limited resection, have been described, including pancreas-sparing duodenectomy, segmental duodenectomy, and local resection. This is based on the clinical experience with gastric GIST, whereby limited wedge resections as opposed to formal gastrectomies have become widely accepted [52,54]. Similarly, limited operative procedures ideally could be the treatment of choice for duodenal GIST. However, unlike the stomach, adequacy of margins and oncologic clearance is a real concern for duodenal GIST. Particularly, given the complex anatomy in the region of the duodenum, resection of duodenal GIST mandates a carefully planned approach via and whether the GIST is limited to the antimesenteric vs. the mesenteric border [1,7].

Limited resection is considered as a treatment option for relatively small duodenal GIST when technically feasible. Wedge resection with primary closure of the duodenal wall can be performed for small lesions (<1 cm) if the resulting lumen is adequate, and the ampulla of Vater can be preserved [10,11]. Segmental resection of the duodenum with the need of a duodenojejunostomy is another possibility for larger tumors arising in the third and fourth portion of the duodenum [12,13]. Some authors have proposed resection and anastomosis even for lesions close to the papilla by performing the anastomosis just below the ampulla [55]. This has been achieved by performing a lateromedial anastomosis opposite to the papilla or by performing papilloplasty and inserting a temporary stent catheter into the papilla to avoid stenosis following anastomosis close to the papilla [55]. A partial duodenectomy with Roux-en Y duodenojejunostomy can be feasible for larger tumors involving the antimesenteric border of the second and third portions of the duodenum [14,55]. Major resection, via pancreaticoduodenectomy, is indicated when the tumor is located in the first or second part of the duodenum and involves the papilla, pancreas, or the duodenal bulb or if the tumor is large with high malignant potential reaching into adjacent organs [1,21].

Factors associated with an increased likelihood of requiring PD versus LR were larger tumors with higher mitotic count and location in the second portion of duodenum [56,57]. In the current study, patients who underwent PD tended to have larger tumors and tumor with higher mitotic count, whereas tumors located at second portion of duodenum were statistically more likely to require PD. Limited resection is perceived to contribute to a better quality of life since it provides functional preservation of the pancreas and continuity of the gastrointestinal tract [17,30,32]. However, while LR may be simpler to perform or less demanding if performed laparoscopically, there is a risk of subsequent anastomotic leakage or stenosis and perhaps compromise on oncological outcome [1,7,27,32,58,59]. In contrast, PD can provide a wider tumor clearance but may be associated with significant short- and long-term morbidity [56,60], which was not confirmed in our study (*p* = 0.128) but is explainable in that the sample size is limited. LR is preferable to avoid procedure-related morbidity and preserve the patient’s quality of life. This underlines that we have to question the indication for duodenopancreatectomies for submucosal tumors with potentially benign appearance.

Imatinib mesylate (IM) has played a revolutionary role in the management of primary and metastatic GIST. Administration of IM therapy in neoadjuvant setting should be considered in cases of locally advanced GIST located in the second part of duodenum and in patients with larger tumors scheduled for PD [4]. This approach can result in downsizing the duodenal GIST and facilitating the surgical procedure by preserving the normal biliary and pancreatic structures. We followed this approach in our study in patients with duodenal GIST scheduled for PD. By downsizing the tumor, we were able to downstage the surgical procedure from PD to LR in 40% of the cases treated with neoadjuvant IM. However, even if neoadjuvant treatment does not affect the surgical procedure, a neoadjuvant IM therapy should well be considered because PD is expected to be safer when the tumor is smaller. Moreover, preoperative IM treatment tends to reduce fragility of GIST and decrease the risk of tumors rupture. Prospective, non-randomized studies have been conducted to evaluate preoperative imatinib for treatment of locally advanced GIST demonstrating that neoadjuvant IM therapy can play a significant role in tumor shrinkage leading to decrease of the extent of resection (i.e., organ-preserving procedures) and to completeness of resection [61,62,63,64,65]. We recommend neoadjuvant therapy to be used selectively in locally advanced duodenal GIST that are not amenable to LR or preservation of the pancreas, although clinical judgement is necessary to estimate the likelihood of conversion to a LR.

Survival analysis results were obtained for all the 13 patients of our study. OS and DFS rates of our cohort were 92.5% and 91.5% at one year, 84% and 83% at three years, and 73.5% and 72% at five years, respectively. The results are much consistent with studies previously reported [23,26,27,28,36]. The overall five-year survival rate of patients following resection for GISTs ranges from 60 to 100% and the five-year DFS from 44–100% [56,57,60]. However, the largest series of duodenal GIST (*n* = 156) from the pre-imatinib era noted local recurrence, metastasis, or both in 35% of their patients [4]. The reason for the improved outcome for patients with duodenal GIST is probably multifactorial and may be related to earlier presentation, smaller tumors, lower risk classification, and administration of IM therapy.

Factors associated with OS and DFS did include tumor size, mitotic count, risk classification, and IM administration [1,32,34,54,66]. In our study, a trend for better OS and DFS in the subgroup of patients with smaller tumor size (<5 cm) and lower mitotic count (<5/50 HPFs) was observed. Margin status was not associated with OS or DFS since the overwhelming majority of patients had an R0 resection—making margin status difficult to evaluate as a prognostic factor. Besides, IM association did not show any correlation with survival rates since the sample size was small. The type of procedure was also not correlated with OS or DFS as a result of the distribution of the two major risk factors among the two groups, leading to the conclusion that both surgical approaches offer equal or similar oncological results.

The present study has several limitations. First, the sample was small, but duodenal GIST remains a rare tumor. The retrospective design with its inherent biases in patient selection for each type of surgical approach is a further limitation. Besides, the optimal duration and indication of adjuvant IM therapy, especially after neoadjuvant imatinib, has not been established; it is difficult to determine the efficacy of adjuvant imatinib. Furthermore, molecular characteristics were not identified in some patients, and a longer median follow-up was available for the PD group. These points could counter-balance the results. Observations from large multicenter studies or even better prospective randomized clinical trial could offer more comprehensive understanding regarding the management and the recurrence patterns of duodenal GIST.

## 5. Conclusions

Duodenal GIST are a relatively uncommon subset of GIST. The anatomy of the pancreaticoduodenal region poses a surgical challenge for duodenal GIST. LR is a reasonable option for resection of GIST of the duodenum and should be considered whenever technically feasible. Since both limited and extended surgery yield comparable survival rates in experience centers, the type of procedure should be chosen according to the specific duodenal site of origin and tumor size considering the associated disease and the performing status of the patient. Recurrence of disease is primarily dependent on tumor biology, including tumor size, mitotic index, and risk classification rather than surgical approach. The administration of IM in neoadjuvant setting should always be considered, especially in patients who are candidates for PD, because it might facilitate the surgical procedure and increase the chance of preserving normal biliary and pancreatic anatomy and is associated with shorter hospital stay and lower risk of perioperative complications.

## Figures and Tables

**Table 1:** Clinical and histopathological data and follow-up data of the whole cohort and of each group (LR vs. PD).

Characteristics		Total (*n* = 13)	LR Group (*n* = 8)	PD Group (*n* = 5)	
Demographics					
Sex					
	Male	7 (54%)	4 (50%)	3 (60%)	
	Female	6 (46%)	4 (50%)	2 (40%)	
Median age (range)		64 (42–77)	61.25 (42–77)	68.4 (59–74)	
Symptoms					
	Abdominal pain	7	4	3	
	GI bleeding	2	2	0	
	Both	2	1	1	
	Anemia	1	0	1	
	Asymptomatic (discovered incidentally)	1	1	0	
Tumor factors					
Tumor site					
	D2	5 (38.5%)	1	4	
	D3	5 (38.5%)	4	1	
	D4	3 (23%)	3	0	
Median tumor size (cm)		5.2 (1.5–13.3)	4.7 (1.5–13.3)	6.1 (1.8–7)	
Tumor size at diagnosis (cm)					
	<5 cm	4 (30%)	3 (37.5%)	1 (20%)	
	>5 cm	9 (70%)	5 (62.5%)	4 (80%)	
Median mitotic index		15 (0–150)	7.5 (0–50)	34 (1–150)	
Mitotic index					
	<5/50 HPFs	10	7	3	
	>5/50 HPFs	3	1	2	
Risk NIH classification					
	Very low	3	2	1	
	Low	3	0	3	
	Intermediate	3	1	2	
	High	4	2	2	
Risk Miettinen classification					
	No	3	2	1	
	Very low	0	0	0	
	Low	3	3	0	
	Intermediate	0	0	0	
	High	7	3	4	
Surgical data					
R-Status					
	R0	12	8	4	
	R1	1	0	1	
Tumor rupture					
	No	13	8	5	
	Yes	0	0	0	
Operative time		195 (80–465)	140 (80–245)	330 (210–465)	
Outcomes					
Length of hospitalization			8 (5–11)	16 (10–24)	
Morbidity					
	No	12	8	4	
	Yes	2	0	2	
Major complications		1	0	1	
Disease progression		4	2	2	
Neoadj TKI					
	Yes	5	3	2	
	No	8	5	3	
Adj TKI					
	Yes	5	3	2	
	No	8	5	3	

PD, pancreaticoduodenectomy; LR, limited resection; D1, D2, D3, D4 portion: 1, 2, 3, 4 of the duodenum; HPF, high-power field; NIH, National Institutes of Health; TKI, tyrosine kinase inhibitor. Other includes anemia, bleeding (i.e., bloody stools).
